# Prediction and Prevention of Gestational Diabetes Mellitus and Its Sequelae by Administering Metformin in the Early Weeks of Pregnancy

**DOI:** 10.7759/cureus.31532

**Published:** 2022-11-15

**Authors:** V Seshiah, S C Bronson, V Balaji, R Jain, C Anjalakshi

**Affiliations:** 1 Diabetology, The Tamil Nadu Dr. Maruthur Gopalan Ramachandran (MGR) Medical University, Chennai, IND; 2 Diabetology, Institute of Diabetology - Centre of Excellence, Stanley Medical College and Hospital, Chennai, IND; 3 Diabetology, Dr. V. Balaji Diabetes Care Centre and Research Institute, Chennai, IND; 4 Public Health, Jain Hospital, Kanpur, IND; 5 Obstetrics and Gynecology, Institute of Obstetrics and Gynecology, Madras Medical College, Chennai, IND

**Keywords:** primordial prevention, early screening, non-communicable diseases, transgenerational transmission, fetal glucose steal, fetal islet cell, fetal hyperinsulinemia, gdm- gestational diabetes mellitus, diabetes type 2

## Abstract

Diabetes mellitus in recent years has become a relentlessly evolving pandemic. Measures for the screening and early detection of diabetes are practiced all around the world. However, considering the ever-increasing magnitude of the problem, the present efforts should especially focus on the primordial prevention of diabetes.

A ray of hope for preventing the development of diabetes in an individual arises from the concept that many adult-onset diseases have already been programmed while the individual was still in-utero. In women with hyperglycemia-in-pregnancy, maternal hyperglycemia results in fetal hyperinsulinemia, which leads to increased adiposity in the fetus, and insulin resistance and diabetes in adulthood. We have ventured to point out that the fetal beta-cells start secreting insulin at 10-11 weeks of pregnancy and fetal hyperinsulinemia persists with maternal hyperglycemia, in a pregnant woman who would develop gestational diabetes. Considering the fetal glucose-steal phenomenon and the fetal renal threshold for glucose, we have suggested a two-hour post-prandial blood-glucose (PPBG) value of >110 mg/dL as the cut-off for the prediction of gestational diabetes in the early weeks of pregnancy. Furthermore, we have emphasized the use of metformin in addition to medical nutrition therapy in the early weeks to maintain PPBG around 110 mg/dL to prevent gestational diabetes.

In this paper, we recommend early, universal screening of all pregnant women during the early weeks of the first trimester and put forward that a two-hour PPBG of >110 mg/dl during the 8^th^-10^th^ week of pregnancy would predict the risk of gestational diabetes in the pregnant woman. We suggest early testing and intervention to prevent the development of fetal hyperinsulinemia as a primordial prevention approach for diabetes.

## Introduction

Diabetes mellitus is a relentlessly evolving pandemic and a major public health problem in recent decades. Diabetes affects millions of people worldwide with a considerable adverse impact on the quality of their lives. Efforts are underway all around the world to detect diabetes in its early stages and prevent its complications. However, in clinical practice, what is achieved by screening for hyperglycemia and detecting diabetes in the early stages of its natural history, say at the level of impaired glucose tolerance, is akin to primary prevention of diabetes. Similarly, treating a patient with diabetes and managing those with diabetic complications correspond to ‘secondary prevention’ and ‘tertiary prevention,’ respectively.

Detection of diabetes by screening does not mean preventing the disease per se from occurring. In this paper, we try to elucidate why efforts toward a primordial prevention approach are needed, indeed, to prevent diabetes.

## Technical report

Primordial prevention of diabetes - whom to focus on?

In 2001, the Centers for Disease Control and Prevention issued a press statement that the twin epidemics of diabetes and obesity threatened the health of Americans. It also held, based on the results of the Diabetes Prevention Program, that diabetes could be prevented by promoting a healthy lifestyle. However, one could see that in 2001, 6.1% of the US population had been diagnosed with diabetes, and currently about 10.5% of Americans have diabetes [1], leading us to rethink the goals of diabetes prevention.

From the zygote

From a single-cell zygote to a well-formed fetus at confinement, remarkable changes occur due to maternal fuels and hormonal influence on fetal development. The crucial period in fetal development is the first trimester­ [2].

The first trimester begins on the first day of the last menstrual period and lasts until the end of week 12. This means that by the time one knows for sure about her pregnancy, she might already be five to six weeks through her pregnancy. A lot happens during the first three months.

Pre-pregnancy metabolic changes may exert their effects on fetal development through modification of oocyte metabolism, predominantly at the level of the mitochondria. Abnormalities in mitochondrial metabolism in the oocyte are likely to predispose to the development of obesity and insulin resistance, thereby contributing to the transgenerational programming of metabolic diseases [3].

At fertilization, it is only the nucleus of the spermatozoon that enters the ovum, and thus, all the cytoplasm, mitochondria, and mitochondrial DNA are exclusively maternally inherited. Therefore, as Kingo said, 'the female gender is the key to diabetes prevention’ [4]. A baby with low birth weight or one who is large-for-gestational age at birth carries an increased risk of obesity, diabetes, hypertension, and cardiovascular disease in adult life. When the offspring is a girl, the transgenerational transmission continues [2,4].

Intra-uterine programming

The famous British epidemiologist David Barker pointed out that an individual’s susceptibility to developing lifestyle diseases in adulthood had already been programmed while he/she was still in-utero. This, he called the ‘fetal origins of adult disease’ (FOAD) hypothesis. Genetics, epigenetics, and environment, all play a significant role in the developmental origins of disease. A susceptible individual might inherit unfavorable genes from his parents; his genes might undergo adverse epigenetic influences in-utero; he might be exposed to an unhealthy postnatal environment in his childhood and adulthood [2]. ‘Genes load the gun and environment pulls the trigger' is a well-known aphorism.

‘Gestational programming’ is a process in which adverse stimuli such as excess maternal fuels or any stresses that occur during sensitive periods of fetal growth, permanently change the structure, physiology, and metabolism, thereby predisposing the individual to an increased risk of diseases in adulthood [5].

Exposure of the fetus to a hyperglycemic environment in-utero is associated with an increased risk of developing impaired glucose tolerance and having decreased insulin secretion in adulthood. This risk is independent of the genetic predisposition to type-2 diabetes mellitus (T2DM) that the child inherits from its parents [6]. Embryos from euglycemic Wistar rats with a low genetic risk for diabetes, when grown in the hyperglycemic intra-uterine environment of Goto-Kakizaki rats, developed into offspring that were significantly more hyperglycemic at six months of age. Furthermore, the Pima Indian offspring who had been in-utero when their mothers had diabetes had a greater risk of diabetes in their life than earlier siblings born before the mother developed diabetes [7].

The fetal pancreas and its handling of glucose

The human pancreas begins to develop four weeks after conception, and first insulin deposits can be found between weeks seven and eight. Pancreatic islets differentiate at the 10th & 11th weeks of gestation and recognize and respond to maternal glycemia from the 11th week of gestation. Each islet functions as an endocrine organ [8]. The central nervous system and heart are among the most sensitive organs and are prone to be affected by in-utero insults in the embryonic stage due to abnormal metabolism.

**Figure 1:**
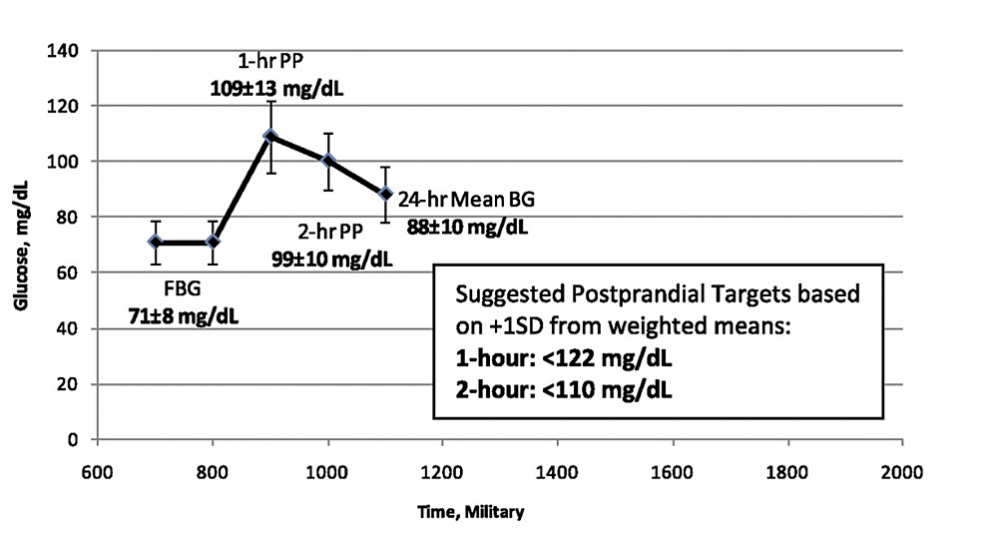
Pattern of glycemia in normal pregnancies and recommended therapeutic targets for post-prandial blood glucose in hyperglycemia-in-pregnancy (HIP). FBG- fasting blood glucose; PP- post-prandial blood glucose; Mean BG- mean blood glucose; SD- standard deviation (Adapted from Hernandez et al., 2011 [9], under the Creative Commons License http://creativecommons.org/licenses/by-nc-nd/3.0/)

In a pooled analysis of 12 studies, involving 45 years of data, Hernandez et al., studied the pattern of glycemia in normal pregnancy and showed that the glycemic targets in the management of hyperglycemia-in-pregnancy (HIP), need to be lower than the currently used ones. They observed the normal levels of fasting blood glucose (FBG) to be 71±8 mg/dL (3.9±0.4 mmol/l) and post-prandial blood glucose (PPBG) to be 99±10 mg/dL (5.5±0.6 mmol/l). They have suggested therapeutic PPBG targets of <122 mg/dL (6.8 mmol/l) at one hour and <110 mg/dL (6.1 mmol/l) at two hours (Figure 1) [9].

Furthermore, Hinkle et al. showed that HbA1c levels measured at 8-13 weeks in pregnancy were significantly higher (an average of 5.3% [34mmol/mol]) in women who developed gestational diabetes mellitus (GDM) later, than those who did not develop GDM (an average of 5.1% [32mmol/mol]). Each 0.1% rise in HbA1c beyond 5.1% in early periods of pregnancy was found to be associated with a 22% increase in the risk of GDM [10].

Significance of the fetal renal threshold

The renal threshold for glucose in the fetus is around 110 mg/dL. When the maternal glucose level is >110 mg/dL, it causes fetal glycosuria. Therefore, uncontrolled maternal diabetes is associated with polyhydramnios from fetal polyuria [11].

Fetuses are exposed to increased amniotic fluid glucose before 12 weeks of gestation, suggesting that metabolic perturbations are underway before diagnosis and that earlier screening and intervention may be warranted.

Fetal glucose steal phenomenon

Poor glycemic control early in pregnancy would result in maternal hyperglycemia, leading to fetal hyperglycemia, and in the establishment of resultant fetal hyperinsulinemia, causing an exaggerated fetal glucose steal.

Consequently, the overactive glucose steal would increase the disposal of maternal glucose into the fetus, thus attenuating the levels of maternal hyperglycemia. Importantly, this effect of lowering maternal glucose driven by the fetus would be greatest in pregnancies with the most hyperinsulinemia fetuses.

Fetal glucose steal is a lesser emphasized phenomenon in diabetic pregnancies. An exaggerated glucose steal by a hyperinsulinemic fetus could also attenuate maternal glucose levels during an oral glucose tolerance test (OGTT), explaining why some mothers with affected fetuses have a seemingly normal glucose tolerance. 

Thus, there is a risk that GDM might not get detected in women with hyperinsulinemic fetuses, especially in the early period of pregnancy [12].

Fetal handling of maternal glucose and tackling the issue by early intervention

Hinkle et al. in 2018 [10] pointed out that an HbA1C of 5.3 in the 10th week predicts GDM (correlating to blood glucose [BG] >110 mg/dl). However, so far, to the best of our knowledge, no explanation has been given for this prediction of GDM. We venture to suggest that fetal beta cells start secreting insulin around the 10 -11th week of pregnancy [8], and in patients with HIP, once fetal beta cells start secreting insulin, fetal hyperinsulinemia persists with maternal hyperglycemia.

If PPBG at the 10th week is > 110 mg/dL, BG has to be brought to < 110 mg/dl before the 11th week as fetal beta-cells start secreting insulin at that time. With fetal insulin secretion, changes in maternal glucose metabolism begin. Consequently, hyperglycemia and hyperinsulinemia promote fetal adiposity and result in impaired glucose tolerance and T2DM in adulthood [2].

Fetal hyperinsulinemia would favor a persistently high glucose flux even at times when maternal blood glucose is normal. The obvious implication is that glycemic control needs to be optimized very early in pregnancy to prevent the establishment of fetal hyperinsulinemia.

## Discussion

Therefore, considering the fetal handling of maternal glucose and the fetal renal threshold for glucose, prediction of GDM can be done if the two-hour PPBG is >110 mg/dL at the 10th week. Hence, in the 8th week itself, PPBG needs to be measured. In case the PPBG is >110 mg/dl, a grace period of two weeks is available to maintain PPBG <110 mg/dl at 10-11 weeks, to prevent the development of fetal hyperinsulinemia. If PPBG is >110 mg/dL, medical nutrition therapy and a low dose of metformin, say 250 mg twice a day, may be started and continued till the end of the first trimester. The target glycemia to be obtained is PPBG 99 ± 10 mg/dL [9]. Furthermore, an intervention by using metformin is safe as the embryonic stage is completed by the 8th week [13]. Metformin decreases insulin resistance and improves insulin sensitivity, thereby decreasing the excessive maternal glucose flux to the fetus. This in turn serves to prevent fetal hyperinsulinemia and excess adiposity in the fetus. Furthermore, in a recent analysis [14] of a large data set of pregnancies exposed to metformin, it was observed that except for an increased risk for small-for-gestational-age infants, maternal exposure to metformin during pregnancy was not found to be associated with any long-term increased risk of hypoglycemia, hyperglycemia, obesity, diabetes, or problems in motor-social development, when compared with insulin. This register-based cohort study has added evidence to the positive benefit-risk balance for the use of metformin in pregnancy [14]. 

Sub-categorization of glucose intolerance in pregnancy

The usual recommendation for the detection of GDM is to perform an OGTT at the 24th-28th weeks of pregnancy [15]. Whereas as we saw above, prediction of GDM can be done in early pregnancy itself if the HbA1C is >5.3% or a two-hour PPBG is >110 mg/dL at the 10th week.

The fetal beta cell starts secreting insulin around the 10th-11th weeks of pregnancy. We hypothesize that, in a woman with HIP, once fetal beta cells start secreting insulin, fetal hyperinsulinemia persists with maternal hyperglycemia.

As the pregnancy progresses, several hormonal interplays occur and result in insulin resistance. Now maternal insulin secretion tends to increase to overcome the increased resistance. But in women who are not able to compensate, GDM ensues. 

Therefore, there is a need to lower the cut-off to detect glucose intolerance, especially in the early weeks of pregnancy. With this background, we put forward a practical sub-categorization of glucose intolerance in pregnancy (Table 1).

**Table 1:** Sub-categorization of glucose intolerance in pregnancy. GDM- gestational diabetes mellitus; GGI- gestational glucose intolerance; EGGI- early gestational glucose intolerance; BG- blood glucose; T2DM- type-2 diabetes mellitus

Category	Cut-off values	Associated risks
GDM	2-hour BG ≥140 mg/dL	Adverse pregnancy outcome and future T2DM
Gestational Glucose Intolerance (GGI) [16]	2-hour BG >120 mg/dL and <140/dL	Adverse Pregnancy Outcome and Future T2DM
Early Gestational Glucose Intolerance (EGGI) (8^th ^- 12^th^ weeks)	2-hour BG >110mg/dL	Prone to develop GDM

The merit of this paper is that it reports an early means to recognize, predict, and prevent gestational diabetes in a woman and future diabetes in the offspring, considering the physiological processes like fetal islet function and renal threshold. The limitation is that though we have analyzed previous observations and postulated the inferences of this paper, further large-scale studies would add more evidence and support to this concept.

## Conclusions

The risk of diabetes to an individual is contributed by his intra-uterine milieu, genes, and external environment. By targeting the risk conferred by the intra-uterine adverse milieu, that is fetal hyperinsulinemia, the risk of obesity and diabetes can be reduced to a considerable extent. Ideally, to achieve this effect, the target BG levels should be closer to that of normal pregnancy: FBG- 80-90 mg/dL; PPBG- 110-120 mg/dL (Mean BG-95 to 105 mg/dL). The goal is to obtain newborn babies’ birth weights appropriate for gestational age between 2.5-3.5 kg. 

By this paper, we recommend early, universal screening of all pregnant women during the early weeks of the first trimester and put forward that a two-hour PPBG of >110 mg/dl during the 8th-10th week of pregnancy would predict the risk of gestational diabetes in the pregnant woman. We suggest early testing and intervention to prevent the development of fetal hyperinsulinemia as a primordial prevention approach for diabetes. For pregnant women with gestational diabetes, we emphasize that preventive measures against diabetes and other non-communicable diseases should start from the pre-conception period and confinement and continue throughout life. This includes a healthy lifestyle, a healthy diet, and maintaining an ideal body weight. We also hope that this paper would trigger more research from the scientific community on the concept of primordial prevention of diabetes.

Finally, we would like to reiterate that major developmental events in the natural history of non-communicable diseases begin in-utero. Hence, to achieve a diabetes-free generation, we need to focus on the fetus for the future.
